# Evaluating the role of ChatGPT in rehabilitation medicine: a narrative review

**DOI:** 10.3389/fdgth.2025.1618510

**Published:** 2025-10-29

**Authors:** Meng Luo, Zhaoyuan Duan, Jing Gao, Yalei Sun, Lidian Chen, Xiaodong Feng

**Affiliations:** ^1^ Department of Rehabilitation Center, The First Affiliated Hospital of Henan University of Chinese Medicine, Zhengzhou, China; ^2^ The First Clinical Medical School, Henan University of Chinese Medicine, Zhengzhou, China; ^3^ School of Rehabilitation Medicine, Henan University of Chinese Medicine, Zhengzhou, China

**Keywords:** ChatGPT, rehabilitation medicine, artificial intelligence, clinical decision support, patient education, multi-agent systems

## Abstract

Chat Generative Pretrained Transformer (ChatGPT) has emerged as a sophisticated artificial intelligence (AI) language model in healthcare. This narrative review examines ChatGPT's current applications and limitations in rehabilitation medicine through analysing multiple studies. While demonstrating promising performance in structured tasks and basic medical guidance, significant challenges persist. These include inconsistent performance in complex clinical scenarios, limited regional adaptability, poor reference reliability, and inadequate safety considerations for special populations. Although innovative approaches like multi-agent systems show potential improvements in accuracy and interpretability, concerns regarding clinical responsibility, data security, and ethical implications remain crucial. As ChatGPT continues to evolve, its optimal integration into rehabilitation practice requires careful consideration of these limitations and appropriate professional oversight. This review aims to provide insights for healthcare professionals and policymakers in navigating the implementation of AI assistance in rehabilitation medicine, emphasizing the need for balanced integration while maintaining clinical safety and effectiveness.

## Introduction

Since OpenAI released ChatGPT 3.5 at the end of 2022, it has attracted worldwide attention and experimentation with its use in various fields (1–3). Now that almost two years have passed, to what extent has ChatGPT been used in the field of rehabilitation, and has it become a powerful therapist's assistant? Will ChatGPT really revolutionize rehabilitation medicine, or is it just a promising idea? It is time for a critical answer.

This review encompasses a wide range of studies that have examined ChatGPT's performance in the following areas: medical licensing exams relevant to rehabilitation professionals, clinical reasoning and diagnosis in the context of rehabilitation, provision of patient education and self-management guidance, development and validation of rehabilitation assessment tools, and support for clinical decision making across rehabilitation subspecialties. Exploring these different aspects provides a clear picture of the current capabilities, limitations, and potential of ChatGPT in rehabilitation. This is timely and necessary to critically evaluate a rapidly evolving technology that may have a significant impact on patient care, clinical efficiency, and the future direction of rehabilitation medicine.

In addition, we review current ethical considerations and practical challenges faced by researchers in integrating AI technologies such as ChatGPT into rehabilitation practice, which are critical for practitioners, researchers and policy makers. Therefore, this review aims to provide healthcare professionals and policymakers with critical insights into the current applications, limitations, and potential of ChatGPT in rehabilitation medicine. By systematically analysing existing studies, we seek to support balanced and evidence-based integration of AI tools like ChatGPT into rehabilitation practice, while emphasizing the importance of clinical safety, ethical considerations, and professional oversight.

## Rehabilitation examinations and specialized assessments

**Figure 1:**
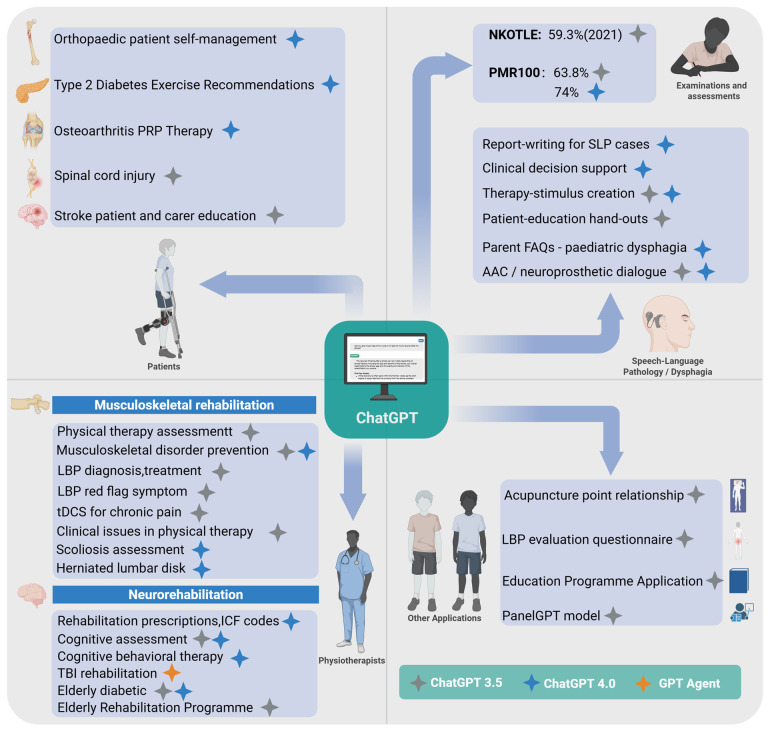
ChatGPT in the field of rehabilitation. Created using BioRender.com.

The approach to therapist qualification examinations varies globally. Taking the U.S. National Physical Therapy Examination (NPTE) as an example, this exam spans areas from foundational sciences to clinical application. The pass rate is closely linked to candidates' academic backgrounds, GRE scores, and other factors (4), with an average first-time pass rate of 84.2% over the past five years (5). Against this backdrop, ChatGPT has demonstrated varying levels of performance across professional qualification exams. In the Korean National Occupational Therapy Licensing Examination (NKOTLE), ChatGPT 3.5 exhibited a steadily improving trend, rising from a pass rate of 52.2% in 2018 to 59.3% in 2021. Although it did not meet the passing standard, it performed well on anatomy and physiology questions, which are less influenced by cultural factors, and displayed strong multilingual adaptability (Figure 1) (6). In comparative studies of more advanced versions, ChatGPT 4.0 demonstrated a clear advantage over version 3.5 in tests with the American Physical Medicine and Rehabilitation Board's PMR100 questions, achieving overall accuracy rates of 74% and 63.8%, respectively, with a notable improvement in response consistency (66.7% vs. 32.2%). Particularly in musculoskeletal system questions, ChatGPT 4.0 achieved an impressive 85.2% accuracy (7). Although ChatGPT exhibits near-human proficiency in general medical knowledge and cross-linguistic applications, further refinement is necessary for managing region-specific legal, policy, and cultural inquiries.

ChatGPT's performance on standardized examinations is a direct reflection of its core capabilities. Its high scores on knowledge-based tests like the PMR100 are achievable because such exams reward the memorization and synthesis of vast medical facts, a task for which large language models are optimized due to their extensive training data. The performance gains with version updates further prove this point. However, its struggles with region-specific legal, policy, and cultural inquiries expose a fundamental deficit: its knowledge is globalized, not localized. This indicates that while AI can master a universal body of medical knowledge, it lacks the contextual background and cultural adaptability essential for integration into a specific professional environment—a critical gap to bridge before it can become a reliable professional aid.

## Clinical reasoning and professional competency assessment

### Medical professional issues – musculoskeletal rehabilitation

Physical therapy assessment constitutes a critical foundation for formulating effective treatment plans and evaluating therapeutic outcomes, where standardization and accuracy are essential for clinical efficacy. While ChatGPT 3.5 displays systematic structure in describing primary assessment components—including patient history, diagnosis, and treatment—it significantly lacks depth and completeness, especially regarding reassessment (only included in 70% of responses) and subjective examination (present in merely 60%) (8). Such deficiencies undermine the iterative clinical reasoning process fundamental to effective musculoskeletal rehabilitation. Further critical evaluation of ChatGPT's clinical applicability has highlighted scenario-dependent performance. While ChatGPT demonstrates sufficient diagnostic accuracy and clinical reasoning in straightforward cases, it significantly underperforms in complex clinical scenarios that involve ambiguous or nuanced conditions, often producing overly generic, non-specific, and insufficiently tailored treatment recommendations (9). This limitation critically restricts its utility as a reliable decision-support tool in complex musculoskeletal rehabilitation contexts. Conversely, ChatGPT shows acceptable reliability in delivering primary prevention advice for common musculoskeletal disorders. CLEAR tool evaluations confirm that both ChatGPT 3.5 and 4.0 effectively handle general issues such as low back pain, fractures, and knee osteoarthritis, with ChatGPT 4.0 demonstrating marginal improvements in completeness, appropriateness, and relevance (10). Nevertheless, this strength is largely restricted to general preventive scenarios; substantial improvement in clinical precision and sophisticated reasoning is required to support nuanced, patient-specific musculoskeletal rehabilitation strategies effectively.

The clinical assessment of lower back pain (LBP) epitomizes the complexities inherent in musculoskeletal rehabilitation, where comprehensive evaluation critically distinguishes benign, self-limiting conditions from severe pathologies necessitating urgent intervention (11). Evaluations based on the 2020 North American Spine Society (NASS) guidelines reveal significant variations in ChatGPT 3.5's performance: achieving acceptable accuracy (72%) only in scenarios clearly supported by guidelines (baseline 65%), but substantially declining to 58% (baseline 46%) in areas of conflicting or limited evidence, and alarmingly dropping to 16% (baseline 49%) when robust evidence is lacking (12). This marked disparity underscores ChatGPT's heavy reliance on explicit guideline-based recommendations and illustrates critical limitations in its capacity to manage ambiguity in clinical decision-making. ChatGPT shows commendable accuracy in identifying critical red-flag symptoms—an essential component in LBP management. In a mixed-method evaluation involving 70 clinical scenarios (58 single-symptom, 12 multi-symptom), ChatGPT 3.5 achieved high relevance and completeness scores, consistently including critical health warnings in 93.1% of single-symptom and 100% of multi-symptom cases (13). Nonetheless, despite its strength in standard presentations, its capability to recognize subtler or atypical clinical manifestations remains uncertain and merits cautious interpretation. Further exploring ChatGPT's clinical reliability, researchers compared its recommendations with established clinical guidelines in complex interventions such as transcranial direct current stimulation (tDCS) for chronic pain. Despite generally valuable insights, frequent deviations from guideline recommendations highlight potential clinical risks, emphasizing the need for caution and expert oversight in applying ChatGPT-generated advice to technically sensitive clinical interventions (14). Crucially, further studies reveal substantial credibility issues concerning ChatGPT-generated references. Despite moderate content accuracy (median: 3.0), the verification rate of its citations is notably low (only 18.9%), severely limiting its reliability in evidence-based practice where precise source verification is imperative (15). In summary, ChatGPT's performance in musculoskeletal rehabilitation scenarios consistently reveals critical shortcomings in complex clinical reasoning and precision-demanding contexts, reflecting intrinsic constraints related to its algorithmic nature and limitations in synthesizing multifaceted clinical data for advanced decision-making.

Spine-related disorders constitute a challenging and significant area in musculoskeletal rehabilitation, given their complexity and clinical variability. For instance, scoliosis severity assessment typically relies on radiographic Cobb angle measurements; thus, accurate interpretation and classification profoundly affect clinical decision-making and patient outcomes. Recent studies indicate ChatGPT 4.0 has achieved high classification accuracy for scoliosis, showing a 100% concordance rate in diagnostic classification across 56 single-curve cases (16). Moreover, despite this accuracy, ChatGPT 4.0 notably fails to fully adhere to established scoliosis classification criteria, demonstrating particular inaccuracies in identifying milder cases with Cobb angles below 10°, a clinically critical threshold where precise differentiation significantly impacts treatment planning. A similar limitation emerges in clinical applications for disc herniation. Evaluations indicate that while ChatGPT 4.0 provides accurate information in 75% of common patient queries—91.7% considered safe, and 75% practically useful (17) — these findings predominantly reflect routine clinical queries rather than more complex, individualized therapeutic scenarios. Critically, when confronted with complex clinical decisions, such as nuanced treatment planning or interpretation of specific medical data and imaging results, ChatGPT demonstrates significant shortcomings. Its responses frequently lack the detail, individualisation, and integrated clinical judgment necessary for effective clinical practice, underscoring the need for cautious use in these contexts. Overall, the pattern of ChatGPT's performance across various musculoskeletal conditions highlights a consistent limitation: while capable in straightforward clinical scenarios, it repeatedly fails to achieve satisfactory outcomes when confronted with complex, precision-demanding clinical reasoning tasks. This critical gap likely originates from the intrinsic complexity of musculoskeletal rehabilitation, requiring multifaceted reasoning, contextual sensitivity, and advanced clinical judgment—areas where ChatGPT currently remains notably limited.

The duality in performance observed in this domain stems from the model's fundamental nature. On one hand, its powerful pattern-recognition capabilities allow it to excel in structured, guideline-driven tasks, such as identifying red-flag symptoms for low back pain. On the other hand, it lacks true clinical reasoning and an underlying comprehension of pathophysiology, which causes it to falter in complex, ambiguous scenarios that demand flexible judgment. Its tendency to generate generic advice and unreliable citations is a direct product of its function as a language predictor, designed to produce plausible text rather than to engage in rigorous clinical thinking and fact-verification.

### Medical professional issues — neurorehabilitation

Neurorehabilitation, a crucial component of clinical rehabilitation, poses unique clinical challenges and thus offers significant exploratory opportunities for ChatGPT's integration. In stroke rehabilitation, ChatGPT 4.0 has shown potential utility in formulating rehabilitation prescriptions and assigning ICF codes, particularly within routine medical management and preliminary rehabilitation planning (18). Nevertheless, its clinical utility is markedly constrained in managing chronic stroke phases and accurately predicting long-term prognosis, reflecting substantial limitations in handling prognostic uncertainty and integrating patient-specific longitudinal data. Given the global trend of population aging and the consequent increase in cognitive impairments such as mild cognitive impairment (MCI) (19), the accuracy and reliability of ChatGPT in cognitive assessment—particularly relative to established clinical tools—have become critical areas requiring rigorous scrutiny and validation in neurorehabilitation contexts. Comparative studies employing conventional cognitive assessment tools (MMSE, MoCA) reveal that GPT 4.0 aligns closely with clinician evaluations among cognitively healthy individuals, notably in memory assessment, exhibiting minimal discrepancies (20). However, caution must be exercised in generalizing these promising results to clinical populations, where cognitive deficits are typically more heterogeneous and nuanced. Critically, GPT 3.5 demonstrates significant divergence from clinician judgment in language assessments for stroke patients (*P* = 0.002), highlighting a fundamental challenge for AI-based cognitive evaluation tools. Although optimization of interaction protocols and refined scoring criteria may partially address these disparities, inherent limitations in contextual interpretation and linguistic nuances remain significant barriers to clinical adoption. In cognitive behavioral therapy (CBT) contexts, ChatGPT 4.0 achieved moderate success (44/60) in standardized tasks aimed at the “catch, check, and change” cognitive framework, indicating a degree of capability in identifying and addressing maladaptive thinking patterns (21), Nevertheless, its utility substantially diminishes when handling complex, individualized psychological cases, underscoring the persistent necessity for human therapeutic expertise, especially in contexts requiring emotional insight, interpersonal skills, and adaptive intervention strategies. In traumatic brain injury (TBI) rehabilitation, researchers have innovatively explored multi-agent systems built around GPT 4.0, deploying five specialized agents addressing clinical guideline classification, query retrieval, match assessment, intelligent Q&A, and outcome evaluation with citations (22). Although this approach has notably improved accuracy (3.8 vs. 3.2), interpretability (2.79 vs. 1.07), and empathy (2.63 vs. 1.08) compared to GPT 4.0 alone, the significantly increased response latency (54.05 vs. 9.66 s) (23). This presents practical implementation challenges, raising questions about its real-world feasibility in fast-paced clinical rehabilitation settings. Presently, despite its emerging utility, ChatGPT remains best suited as an adjunctive support tool within neurorehabilitation rather than a replacement for experienced clinicians. The multi-agent system approach merits further exploration, yet critical enhancements in clinical reasoning, individualization, and efficiency are essential prerequisites for broader clinical adoption and genuine advancement of rehabilitation practice.

In the field of rehabilitation for elderly patients, with elderly diabetic patient management as an example, studies comparing GPT 4.0 Turbo and GPT 3.5 in bilingual Japanese-English settings reveal that, while GPT 4.0 provides concise responses, it excels in handling complex tasks; by contrast, GPT 3.5's responses tend to be verbose and often fail to fully consider the specific needs of elderly patients. In practical scenarios such as insulin injection guidance, GPT 4.0 delivers comprehensive step-by-step instructions, yet remains somewhat lacking in safety considerations tailored to older adults (24). In the broader context of rehabilitation program development for elderly patients, researchers, through systematic parameter optimization and expert evaluation, have validated ChatGPT's capacity to generate multidimensional rehabilitation plans encompassing physical therapy, occupational therapy, and speech therapy (25). These studies underscore ChatGPT's advantages in geriatric rehabilitation, such as convenience, personalization capabilities, and potential to reduce the burden on human resources. However, they also highlight its limitations, including constraints in accuracy and a lack of citation support. Thus, while ChatGPT demonstrates considerable promise in geriatric rehabilitation, particularly in delivering personalized care in resource-limited settings, it should currently be regarded as an adjunct tool for healthcare professionals, with further clinical validation needed to ensure its safety and efficacy.

The complexity of neurorehabilitation further amplifies the AI's limitations. It serves effectively as an information-structuring tool for routine tasks like assigning ICF codes, but it cannot manage the advanced clinical challenges that require longitudinal data integration, prognostic judgment, and deep empathy—tasks far beyond the scope of text prediction. While multi-agent systems offer a valid strategy for improving performance by decomposing tasks to simulate multidisciplinary collaboration, their significantly increased latency highlights a key engineering trade-off: the pursuit of higher-quality decisions must be balanced against the practical demand for computational efficiency in clinical workflows.

### Patient services

In addition to supporting professional rehabilitation practitioners, ChatGPT offers patients access to valuable medical guidance. In home-based rehabilitation guidance, early self-management studies for orthopedic patients indicate that ChatGPT 4.0 performs well in accuracy (79.8%), applicability (75.2%), comprehensiveness (70.6%), and clarity of communication (75.6%), particularly excelling in specific scenarios such as managing complications following pediatric forearm fractures (26). For chronic disease management, studies on exercise recommendations for patients with type 2 diabetes reveal that ChatGPT 4.0 provides 71.4% fully accurate and comprehensive information, with strong ratings in safety and practicality, though improvements are needed in the completeness of certain specific recommendations (27). Regarding information on specific treatment options, such as PRP therapy for osteoarthritis, research demonstrates that while ChatGPT 4.0 surpasses version 3.5 in information quality and citation inclusion (56% of responses contain source links), both versions produce content with readability levels that exceed the general public's comprehension (28). In the domain of complex disease information, findings on spinal cord injury are varied: a study based on GPT 3.5 noted significant issues in information quality and readability, requiring a university-level educational background for full comprehension (29). Conversely, another study found ChatGPT's reliability and usability to be higher in specific areas like “complications” and “treatment” (scoring 5.38 and 5.87, respectively) but relatively weaker in “general information” (30). In managing educational needs for stroke rehabilitation patients and their caregivers, ChatGPT received a satisfaction rating of 65.8% compared to Google Bard's 75.8%, with particular room for improvement in addressing safety and emotionally sensitive issues (31). Collectively, these studies suggest that while ChatGPT shows potential in delivering healthcare information, it still faces notable shortcomings in information completeness, readability, and empathetic resonance. These limitations are echoed in low back pain education, where ChatGPT-4.0 demonstrated superior guideline adherence and response quality compared with version 3.5, yet both models underperformed in addressing psychosocial concerns such as anxiety and family support (32). Taken together, such findings reinforce that ChatGPT is best positioned as an adjunct to professional medical consultation rather than a replacement.

In patient services, ChatGPT's performance clearly delineates the boundary between knowledge provision and effective communication. As a powerful knowledge base, it can accurately provide factual information. However, its generally poor readability and lack of empathy are direct consequences of its design optimization, which prioritizes comprehensive and grammatically correct information over accessible and warm delivery. Lacking an emotional model, it cannot truly understand or respond to the nuanced psychological and emotional needs of patients, a limitation that underscores the irreplaceable value of human clinicians in providing care and connection.

### Speech-language pathology applications

New evidence from speech-language pathology (SLP) reinforces that generative models can already relieve clinicians of low-value tasks while still falling short in highly individualised therapy work. In a task-level experiment, Birol et al. evaluated ChatGPT-4 in six core activities and found “high” accuracy for report writing and decision support, but only “medium” performance when the model was asked to craft nuanced therapy stimuli or full session plans—especially for Turkish-language prompts (33). A nationwide perceptions survey conducted while most respondents were using the default GPT-3.5 web interface echoed this mixed picture: more than two-thirds of clinicians and graduate students believed AI would streamline documentation and patient-education hand-outs, yet fewer than one-fifth had deployed it at the bedside, citing confidentiality, cultural fit and the need for explicit institutional guidance (34). Together these data suggest that ChatGPT, particularly in its GPT-4 iteration, is already a credible co-pilot for paperwork and early clinical reasoning, but that therapy-specific outputs still require expert vetting and localisation.

Patient-facing studies reveal comparable strengths—and new gaps—when the model is used for dysphagia education, global service design and assistive communication. Alyanak et al. compared ChatGPT-4 with Microsoft Copilot (GPT-4 derivative) and Google Gemini on 25 parent FAQs about paediatric dysphagia; ChatGPT-4 produced the most accurate and reliable answers, whereas Gemini's prose was easiest to read, underscoring a precision-versus-accessibility trade-off that future rehab-specific fine-tuning must reconcile (35). By contrast, Gallano et al.'s Latin-American review and pilot relied on the free-tier GPT-3.5, showing that 73% of auto-generated Spanish rehabilitation activities were clinically feasible after expert review, yet fewer than half required no cultural rewrites, highlighting the importance of region-specific corpora and infrastructure (36). Finally, Bhamidipaty et al. described how both GPT-3.5 and GPT-4 can layer onto augmentative-and-alternative-communication devices and neuroprosthetic pipelines, potentially transforming real-time dialogue for people with severe speech impairment—provided affordability, data privacy and rigorous real-world validation are addressed (37). Collectively, these five studies broaden the review's interdisciplinary scope, illustrating the immediate administrative utility of ChatGPT across SLP settings while pinpointing the cultural, ethical and technical refinements still needed for patient-centred language and swallowing rehabilitation.

Performance gaps between GPT-3.5 and GPT-4 in SLP largely stem from three technical issues. First, GPT-4's larger context window and parameter count allow it to keep track of multi-step clinical reasoning, so it excels in report writing and decision support, whereas GPT-3.5 often truncates key details. Second, SLP terminology (e.g., dysphagia staging, AAC jargon) is poorly represented in general web data; GPT-4's extra medical fine-tuning partly compensates, but both models still struggle with culture- or language-specific prompts. Third, highly individualised therapy-stimulus design demands creative, context-aware content that neither model has seen in quantity, explaining their shared drop in accuracy for that task. These points show that sheer scale improves baseline accuracy, yet domain-specific, multilingual fine-tuning—and clinician oversight—remain essential.

### Other domains

In other domains of rehabilitation medicine, ChatGPT demonstrates varied developmental potential. In knowledge modeling, studies on acupuncture point relationship extraction reveal that a fine-tuned GPT 3.5 model achieved a micro-averaged F1 score of 0.92, markedly outperforming traditional deep learning models and other GPT versions, underscoring the importance of domain-specific fine-tuning (38). Leveraging this specialized knowledge-processing capability, researchers have further explored ChatGPT's application in the development of assessment tools. A low back pain (LBP) evaluation questionnaire created with ChatGPT 3.5 showed promising results in multidimensional pain assessment, though improvement is still needed in social relationship assessment (39). In educational applications, recurrence quantification analysis (RQA) has revealed distinctive linguistic features in personal statements for physical therapist education programs generated by ChatGPT, offering a novel perspective for admissions evaluation (40). More innovatively, the introduction of the PanelGPT model, which simulates multidisciplinary team discussions involving roles such as physical therapists, psychologists, and nutritionists, highlights ChatGPT's potential for round-the-clock support and personalized recommendations (41). This model shows promise in applications across areas like performance analysis, psychological support, and nutrition management, despite ongoing challenges related to emotional accuracy and data privacy (42). These exploratory studies suggest that ChatGPT holds vast potential within rehabilitation medicine, but its optimal application remains contingent on close collaboration with professionals in the field.

Recent studies further illustrate the potential of large language models in enhancing patient experiences through personalized and accessible interventions. For instance, Han et al. demonstrated that a chat-based mobile auditory training program significantly improved hearing rehabilitation outcomes in experienced hearing aid users, highlighting the practical utility of conversational AI in patient-driven rehabilitation processes (43). Similarly, revealed ChatGPT 4.0's efficacy in enhancing postoperative care for cochlear implant patients through improved patient education and continuous support, underscoring the model's strengths in facilitating patient comprehension and adherence to clinical instructions (44). Collectively, these findings emphasize that targeted and interactive use of LLMs can effectively empower patients and potentially improve clinical outcomes, though explicit guidance and careful oversight from clinicians remain critical.

Across these additional domains, three recurrent factors appear to dictate model success. First, domain-specific fine-tuning is decisive: the acupuncture-point extractor achieved an F1 of 0.92 only after rigorous specialty tuning, whereas a baseline GPT-3.5 model performed noticeably worse on the same task. Second, task dimensionality shapes output quality: low-back-pain questionnaires generated from generic prompts captured biomedical dimensions accurately but failed to address social-context items, indicating that large language models still default to the most prevalent patterns in their training data. Third, multimodal and real-time requirements—such as hearing-aid coaching or cochlear-implant after-care—reveal current limitations in continuous feedback loops, emotion recognition and data privacy. Taken together, these observations suggest that, while large language models can match or even surpass traditional approaches in narrowly defined, text-centric subtasks, dependable end-to-end clinical support will continue to hinge on task-specific datasets, multimodal integration and sustained clinician oversight.

## Context of existing rehabilitation AI tools

**Figure 2:**
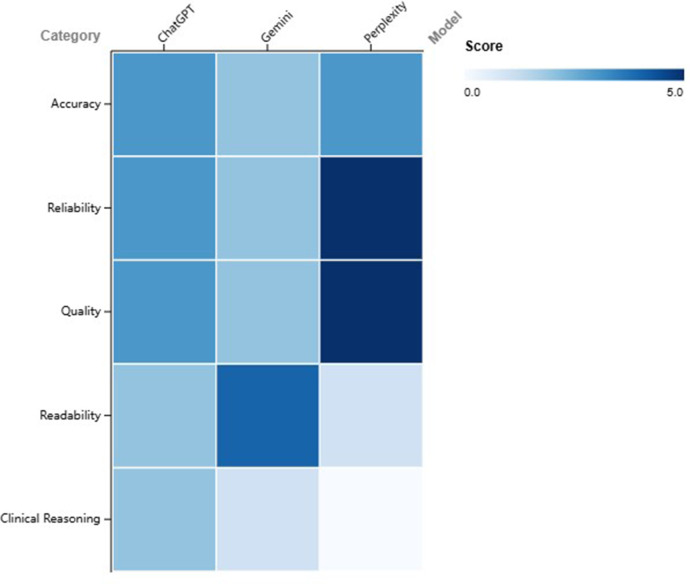
Heatmap comparing the performance of ChatGPT, Gemini, and Perplexity across five evaluation domains. Scores range from 0 (poor) to 5 (excellent), with colour intensity increasing with higher values.

With the growing attention on ChatGPT and the rapid advancement of generative large language models, various models have emerged. Recent studies have comparatively evaluated the performance of ChatGPT, Gemini, and Perplexity within rehabilitation medicine. Investigations into low back pain Q&A have indicated that ChatGPT demonstrates moderate overall accuracy, performing well in treatment and self-management advice but showing high error rates in identifying risk factors and suboptimal readability. Bing and Gemini exhibited similar performance, with ChatGPT-4 slightly outperforming version 3.5 (45). Another study on low back pain found that Perplexity significantly surpassed ChatGPT and Gemini in quality and reliability scores but produced difficult-to-read text. Conversely, Gemini had the best readability, ChatGPT performed moderately, yet faced issues with both quality and readability (46). In the context of vestibular rehabilitation, ChatGPT showed high accuracy in knowledge-based queries but had significant shortcomings in clinical reasoning, whereas Gemini performed even weaker. Expert evaluations identified 25% of ChatGPT's responses as “completely incorrect”, highlighting considerable risk when dealing with complex reasoning tasks (47). Similarly, in a pain-related study, Perplexity again led in reliability and completeness of information but exhibited the lowest readability. ChatGPT was intermediate, generally producing text above recommended patient reading levels (Figure 2) (48).

Collectively, ChatGPT shows acceptable performance in generating basic health information, particularly nearing clinical standards in self-management and treatment recommendations. However, it exhibits substantial weaknesses in risk identification and clinical reasoning, thus carrying risks of misinformation. Perplexity provides more complete and reliable sources but suffers from poor readability, while Gemini offers better readability but lower accuracy and professionalism. Overall, current LLM-based AIs are more suitable as supportive tools rather than primary resources for medical decision-making. Future developments should prioritize improving clinical reasoning capabilities and readability and implement professional verification mechanisms to ensure information safety and efficacy.

The performance differences among various AI models are not accidental but reflect their distinct design philosophies. Perplexity, for instance, builds its core strength on source reliability, enhancing trustworthiness with citations at a cost to readability. In contrast, models like ChatGPT and Gemini are optimized for conversational fluency and naturalness, which results in better readability but carries the risk of generating information that may be inaccurate or unsourced. This trade-off between reliability and readability indicates that no single “best” model exists for clinical use; rather, the optimal tool must be selected based on the specific task, whether it is rigorous evidence retrieval or initial patient communication.

## Dataset development

To move beyond proof-of-concept demonstrations and realise safe, large-scale deployment, a rehabilitation-specific version of ChatGPT must be built on purpose-designed data rather than a generic web corpus. Three complementary streams are essential: multilingual clinical notes and discharge summaries annotated with ICF codes, verbatim therapy transcripts that capture the real-time reasoning of speech-language, occupational and physical therapists, and sensor-linked outcome files—gait-lab metrics, wearable data or audiology logs—that permit multimodal alignment. Practising rehabilitation professionals should be embedded throughout the curation pipeline: they can label red-flag cues, contraindications and culturally specific adaptations, supply counter-examples when the model fails, and vet automatically generated references. A continuous-learning loop that ingests de-identified session data every few months would keep model weights aligned with evolving guidelines and regional practice patterns.

## Clinical integration

Successful clinical integration will depend on a clearly defined human-in-the-loop boundary. A tiered approach is recommended: fully automated information delivery for low-risk tasks such as drafting patient leaflets; supervised AI output—requiring therapist sign-off—for care-plan construction; and real-time co-creation during multidisciplinary rounds only when latency stays below ten seconds. After each chatbot or voice-assistant exchange, patients should be invited to rate relevance and empathy through a one-click interface; aggregated metrics can trigger prompt-engineering updates or focused data augmentation, thereby closing the feedback loop between end users and model developers.

## Policy considerations

Finally, regulatory and institutional policies must keep pace with technical progress. Rehabilitation-oriented LLMs should be mandated to surface verifiable citations for high-stakes content, comply with edge-encrypted inference for sensitive contexts such as paediatric AAC, and operate under a shared-responsibility framework in which vendors guarantee technical accuracy up to a documented confidence threshold while clinicians retain ultimate decision authority. With purpose-built datasets, clinician-guided fine-tuning, and transparent governance, a specialised ChatGPT can evolve from a promising knowledge companion into a context-aware partner that augments—rather than replaces—human expertise in rehabilitation medicine.

## Conclusion

The current applications of ChatGPT in rehabilitation medicine highlight its distinctive performance characteristics across varied scenarios. Results from the NKOTLE examination and PMR100 tests illustrate a marked enhancement in ChatGPT's capabilities with successive versions, particularly reflecting near-human proficiency in foundational medical knowledge and multilingual applications. In clinical practice, ChatGPT demonstrates notable effectiveness in handling structured tasks, such as scoliosis classification and the provision of preventative information for musculoskeletal conditions. Particularly innovative is the adoption of a multi-agent system in neurorehabilitation, which not only improves answer accuracy but also advances interpretability and empathy, providing a promising approach to leveraging ChatGPT in complex clinical environments.
